# A Comparison of Fresh Frozen *vs.* Formalin-Fixed, Paraffin-Embedded Specimens of Canine Mammary Tumors via Branched-DNA Assay

**DOI:** 10.3390/ijms17050724

**Published:** 2016-05-13

**Authors:** Florenza Lüder Ripoli, Annika Mohr, Susanne Conradine Hammer, Saskia Willenbrock, Marion Hewicker-Trautwein, Silvia Hennecke, Hugo Murua Escobar, Ingo Nolte

**Affiliations:** 1 Small Animal Clinic, University of Veterinary Medicine Hannover, Hannover D-30559, Germany; florenza@ripoli.com.br (F.L.R.); annika.mohr@yahoo.de (A.M.); Susanne.Conradine.Hammer@tiho-hannover.de (S.C.H.); Saskia.willenbrock@preclinics.com (S.W.); 2 Hematology Oncology and Palliative Medicine, Clinic III, University of Rostock, Rostock D-18057, Germany; hugo.murua.escobar@med.uni-rostock.de; 3 Department of Pathology, University of Veterinary Medicine Hannover, Hannover D-30559, Germany; marion.hewicker-trautwein@tiho-hannover.de; 4 Institute of Veterinary Medicine, Georg-August-University Göttingen, Göttingen D-37077, Germany; shennec@gwdg.de

**Keywords:** branched-DNA assay, formalin-fixed, paraffin-embedded specimens, fresh frozen tissue, canine mammary tumor, gene expression, RNA

## Abstract

Mammary neoplasms are the tumors most affecting female dogs and women. Formalin-fixed, paraffin-embedded (FFPE) tissues are an invaluable source of archived biological material. Fresh frozen (FF) tissue is considered ideal for gene expression analysis. However, strategies based on FFPE material offer several advantages. Branched-DNA assays permit a reliable and fast workflow when analyzing gene expression. The aim of this study was to assess the comparability of the branched-DNA assay when analyzing certain gene expression patterns between FF and FFPE samples in canine mammary tumors. RNA was isolated from 109 FFPE samples and from 93 FF samples of different canine mammary tissues. Sixteen (16) target genes (*Tp53*; *Myc*; *HMGA1*; *Pik3ca*; *Mcl1*; *MAPK3*; *FOXO3*; *PTEN*; *GATA4*; *PFDN5*; *HMGB1*; *MAPK1*; *BRCA2*; *BRCA1*; *HMGA2*; and *Her2*) were analyzed via branched-DNA assay (b-DNA). *ACTB*, *GAPDH*, and *HPRT1* were used as data normalizers. Overall, the relative gene expression of the two different origins of samples showed an agreement of 63%. Still, care should be taken, as FFPE specimens showed lower expression of the analyzed targets when compared to FF samples. The fact that the gene expression in FFPE proved to be lower than in FF specimens is likely to have been caused by the effect of storage time. *ACTB* had the best performance as a data normalizer.

## 1. Introduction

Mammary neoplasms are the most prevalent neoplasms in female dogs [1] as well as in women [2]. Fresh frozen (FF) tissues are ideal for molecular analysis by gene expression measurements provided their RNA is well preserved. However, such tumor sets are not so abundant [3], and their collection is mainly restricted to tissue banks and research groups. Alternatively, formalin-fixed, paraffin-embedded (FFPE) tissues represent a unique source of archived biological material [4,5] and are the most widely available source of tissue material for which long-term clinical follow-up data are recorded [6]. Strategies based on FFPE material offer several advantages such as easy handling, long-term cheap storage, and suitability for immunohistochemical analyses [7]. The chance to assess gene expression profiling proportioned by the mentioned samples enables the performance of many retrospective and multi-center studies, benefiting the correlation of expression profiles with clinical outcomes [8,9]. However, the RNA of FFPE specimens is of much lower quality than RNA obtained from FF samples [10]. The most remarkable molecular change caused by formalin is the formation of cross-linkages between proteins or between proteins and nucleic acids [11]. Furthermore, several other steps of the paraffin embedding process can affect the RNA [12].

The most common way of assessing canine mammary tumor biomarkers is via immunohistochemical (IHC) evaluation of hormone receptors (ER and PR), which may assist in the approach and prognostic of patients [13]. IHC has proven to be a good technique when performing large multi-center studies, but it is relatively laborious and time-consuming, especially when several target genes are to be analyzed. Considering that the RNA of FFPE samples has some level of degradation and that the yields retrieved may be low, real-time PCR has been the ideal choice to perform gene expression analysis from RNA obtained from those specimens due to the possibility of using fragmented RNA via amplification of the target gene [14]. Nonetheless, the amount of starting material as well as the number of targets to be analyzed are limiting factors in this technology. To overcome this situation, branched-DNA assays (b-DNA) provide the possibility of working with small amounts of samples, as they permit the analysis of up to 500 target genes within a single sample. Additionally, b-DNA assays do not depend on a previous pre-amplification of the nucleic acid (which is a major source of gene-specific measurement errors) nor is it necessary to perform previous purification of the nucleic acid [15]. Moreover, some of the advantages of b-DNA assays when compared to qPCR include a simpler and more rapid workflow and, consequently, decreasing pipetting errors and higher sensitivity [16].

In humans, hormone receptors (ER and PR) are checked for patients who have already been diagnosed with breast cancer (HBC) for predicting response to endocrine therapy [17]. In the UK, women who are at genetic risk of having a defect gene due to family inheritance are offered screening for *BRCA1*, *BRCA2*, and *Tp53* [18]. In canine mammary tumors (CMT), in contrast to the human counterpart, the use of tumor biomarkers is still restricted to research purposes [19]. Therefore, the necessity of analyzing gene expression profiles in different tumor entities to assist in early detection, choice of treatment, and a prognostic assessment of the disease is evident. Based on human breast cancer as well as on other tumor types, 16 genes (*Tp53*; *Myc*; *HMGA1*; *Pik3ca*; *Mcl1*; *MAPK3*; *FOXO3*; *PTEN*; *GATA4*; *PFDN5*; *HMGB1*; *MAPK1*; *BRCA2*; *BRCA1*; *HMGA2*; and *Her2*) are considered to be worth analyzing in dogs given their already known relevance in tumor involvement. Three different housekeeping genes were used as data normalizers (*ACTB*, *GAPDH*, and *HPRT1*).

The hypothesis is, by using the b-DNA assay, that the gene expression of more genes can be measured from a single sample of canine mammary tumor entities. It is also of interest to determine whether the gene expression between FFPE and FF specimens is comparable when applying the referred technique and whether the different storage times have any influence on the outcomes. Moreover, the opportunity to analyze the performance of the different housekeepers in mammary canine tumors is taken.

## 2. Results

### 2.1. RNA Quantification

The RNA yields of the FF samples were clearly more consistent when compared to the ones obtained from FFPE and ranged from 100 to 400 ng, whereas those of FFPE varied from 10 to 1000 ng.

### 2.2. Gene Expression

#### 2.2.1. Housekeeping Genes

The mean expression of the three housekeeping genes (HKG) was calculated in order to achieve an accurate normalization. The results of the FF *vs.* FFPE comparison were normalized against the mean of the HKG (details shown in Table 1). Fifty-seven of 153 (37.25%) genes within the histological groups showed a statistically significant difference.

To assess the different performance of each HKG when comparing FF and FFPE, housekeepers were separated and data were normalized against each one individually. The same procedure as previously mentioned was carried out, and 42 of 153 (27.45%) genes within the histological groups showed a significant difference when data were normalized against *ACTB*. For *GAPDH*, 78 of 153 (50.98%) presented a significant difference, whereas for *HPRT1*, 72 of 153 (47.05%) revealed a significant difference.

#### 2.2.2. Target Genes and Histological Groups

Fifteen FFPE samples were excluded due to the low limit of detection (LOD).

The group of lobular hyperplasia showed *n* = 3 for FF samples and was therefore excluded from the analysis. Considering that it is not possible to establish a comparison of the two groups of lobular hyperplasia between the two different origins of samples, the FFPE group of lobular hyperplasia was not taken into account (*n* = 20).

Ninety-three samples of FFPE for *GATA4* were excluded due to the LOD, whereas 84 FFPE specimens for *HMGA2* were excluded. Sixty-three and 20 samples of FFPE were excluded for *BRCA1* and *BRCA2*, respectively. *Tp53*, *Myc*, and *Pik3ca* had 12 FFPE specimens excluded, respectively. The remaining target genes had a few samples removed, varying from 3 to 9. The results of the target genes and histological groups when comparing FF and FFPE are shown in Table 2.

**Figure 1:**
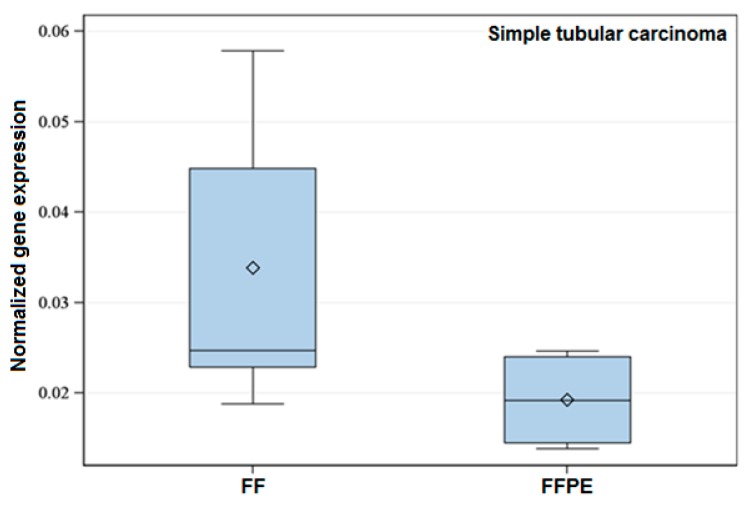
Exemplary box-plot figure showing the normalized expression of *Tp53* within the histological Group 6 (Simple tubular carcinoma). The normalized gene expression of the referred gene is lower in FFPE when compared to FF specimens. Data showed significant difference (*p* < 0.05).

Significant differences were always due to a higher expression of the gene in FF samples (Figure 1) with the exception of the expression of the target gene *PTEN*, which revealed higher expression in FFPE samples.

### 2.3. Effect of Storage Time

**Figure 2:**
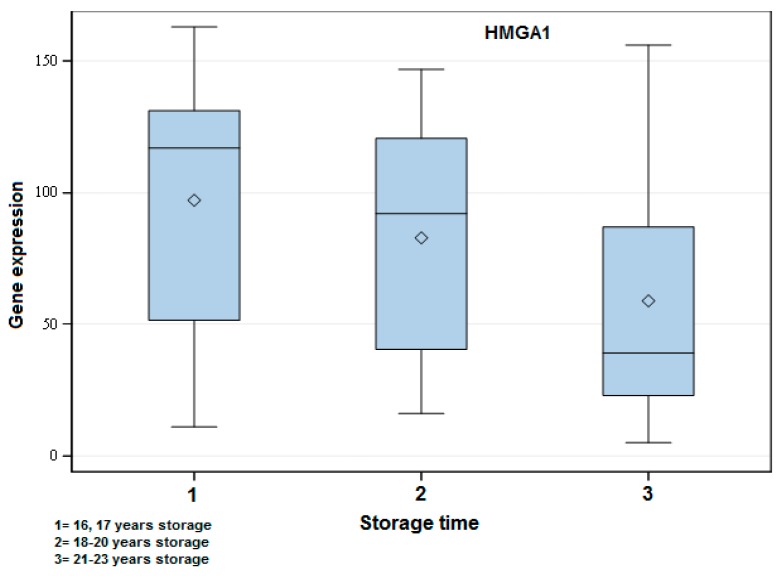
Exemplary box-plot figure showing the gene expression of *HMGA1* when comparing the different storage time groups of FFPE samples. The years of storage are indicated in the figure. There was a significant difference among all groups: 1 *vs.* 2 (*p* < 0.05), 1 *vs.* 3 (*p* < 0.001) and 2 *vs.* 3 (*p* < 0.01). A higher gene expression in the short storage time group followed by Groups 2 and 3 could be observed.

From the target genes showing a significant difference (*p* < 0.05) among the three groups of FFPE samples, *Tp53*, *Myc*, *HMGA1*, *Pik3ca*, *MAPK3*, *FOXO3*, *MAPK1*, *BRCA1*, and *BRCA2* were demonstrated to be clearly overexpressed in the short storage time group (1), followed by the moderate storage time group (2), and last of all the long storage time group (3) (Figure 2). The only exception was *PTEN*, which was shown to be overexpressed in Group 3.

### 2.4. Correlation between Fresh Frozen (FF) and Formalin-Fixed, Paraffin-Embedded (FFPE)

From the target genes, only *FOXO3* and *GATA4* correlated between the two origins of samples (*p* < 0.05). *FOXO3* showed a positive correlation, whereas *GATA4* showed a negative correlation between FF and FFPE.

## 3. Discussion

The aim of the present study was to assess the comparability of the branched-DNA assay when analyzing certain gene expression patterns between FF and FFPE samples stored for many years in canine mammary tumors.

FFPE samples are widely available, as the collection procedures happen as a result of the sampling normally sent to pathology laboratories for diagnostic reasons. Therefore, the availability of such specimens is evident and enables the analysis of a larger pool of cases in a relatively short period of time when compared to FF specimens, which normally offer a limited number of samples [4,6].

To the best of our knowledge, no studies have been performed yet comparing FF and FFPE specimens from canine mammary tumor entities via b-DNA assay. This study suggests that the gene expression from FFPE samples of canine mammary neoplasms is suitable for analysis via branched-DNA assay, and an agreement of 63% to FF samples was reached. However, Knudsen and colleagues showed, when analyzing human xenografts, that the coefficients of reliability (where qPCR analysis of FF was used as control) for FFPE and for FF specimens via b-DNA assay were practically the same [16]. Their results focused on matched pairs of both origins of samples, where the FFPE samples were specifically generated to the referred study (storage time < 72 h). Only four FFPE samples (human tissues) aged between 9 to 13 years old were analyzed in their study, and the b-DNA assay enabled the correct identification of certain genes. Their study had some major limitations when compared to the present study such as a smaller number of samples (*n* = 20 for xenografts and *n* = 4 for human tissues) as well as a smaller number of target genes (*n* = 5). 

In the present study, gene expression in FFPE was lower than in FF specimens, probably due to some degradation effect over time. A comparison among three different storage times of FFPE revealed a clearly higher expression of target genes in the short storage time group with a decrease in the expression in the other groups. This outcome shows that an overtime effect plays a role in the gene expression. Similarly, a study from 2004 showed that fragmentation of RNA continues to occur during storage time for human FFPE specimens [21]. In a more recent study in human medicine, Nam and colleagues reported similar results when analyzing samples via qPCR, where the amplification of the target gene decreased as the storage time of FFPE specimens increased—the target gene was amplified in all samples (100%) stored for less than one month, in 62.5% of samples stored between one month to one year, and in 58.3% of samples stored from three to eight years [22]. It would be ideal to directly compare the storage time of FF and FFPE samples herein to check if other variables apart from time also influenced the lower expression of FFPE when compared to FF specimens. Unfortunately, in this study, this was not possible due to the lack of FF and FFPE samples with the same storage time.

Only two genes (*FOXO3* and *GATA4*) appeared to correlate between FF and FFPE specimens. In contrast, a study from 2011 showed a high correlation between 20 matched paired samples of FF and FFPE, where specimens had been stored for two years [6]. A relevant aspect to be considered and which might explain such discrepancies when comparing both studies is with regard to matched probes as well as the short storage time used in their study in contrast to the present one.

The common way of checking canine mammary tumor biomarkers is via IHC evaluation of hormone receptors (ER and PR) [13]. IHC proves to be a good technique when performing large multi-center studies. However, it is relatively laborious and time-consuming, especially when analysis of several markers is needed [23]. An important limiting factor of this technique when considering the present study is that it does not allow gene expression analyses to be performed [24]. Moreover, a previous study showed that slight but nevertheless significant changes in IHC occurred over time with a decrease in the staining intensity with all storage conditions except for the ones stored at 4 °C [25].

Major limitations of FFPE analyses by qPCR when compared to b-DNA assays are the necessity of previous purification of the nucleic acid, the need for target gene pre-amplification, and higher amounts of required RNA [7]. The RNA retrieved from the FFPE samples herein were considerably low in comparison to the ones obtained from FF, thus limiting qPCR analyses due to the small amounts of starting material. Furthermore, a limited number of targets analyzed per time and a more complex and longer workflow with a consequently higher probability of pipetting errors and lower sensitivity are still disadvantages of qPCR when compared to b-DNA assays [26,27]. Branched-DNA experiments allow rapid and accurate analysis [16] of up to 500 unique targets within a single sample, a fact that was decisive in choosing the method in this study.

The RNA yields of FF samples in this study were typically more consistent than FFPE, presenting, on average, higher RNA yields. Several variables can affect the RNA from the moment of the collection of samples up to the pre-fixation time, the duration of fixation until the final storage of the specimens [12]. Formalin is toxic and may lead to RNA degradation [12]. Masuda and collaborators showed that nearly 40% of the adenine of RNA is affected by formalin; consequently, the cDNA synthesis by reverse transcriptase is compromised. Thereby, the RNA from formalin, fixed samples may lack the target area for an eventual amplification [28]. Interestingly, the high amount of excluded samples, all of which were FFPE, was due to the low LOD for *BRCA1*, *BRCA2*, *GATA4*, and *HMGA2*. The results of the present study suggest that the degeneration described previously might have happened and that the amplification of the signal was in some manner compromised. A limitation herein is that it was not possible to assess the RNA quality of FFPE specimens due to the lack of sufficient RNA material to perform both the quality test and real experiment, thus allowing no comparison between RNA quality of FF and FFPE.

Housekeeping genes (HKG) are usually selected to correct expression data when analyzing gene expression measurements [29]. Their expression is usually stable in different tissues. However, studies showed that they may vary under hypoxia [30] or neoplastic growth [31], which can lead to a misinterpretation of the results. To our knowledge, there are still no commonly accepted HKG for analysis in canine mammary neoplasms. The HKG used in the present study were based on the data normalizers commonly reported in human literature regarding breast cancer [32,33]. Data normalized against *ACTB* presented the lowest significant difference followed by *HPRT1* and *GAPDH*. De Kok and colleagues showed that the best HKG as data normalizers for human breast cancer were, when only taking into consideration the ones utilized here, *HPRT1*, followed by *ACTB* and *GAPDH* [29]. In the present study, the expression of the HKG varied; therefore, the mean expression of them was used as suggested in the literature in an attempt to avoid analysis bias [34].

In HBC, a considerable number of prognostic markers have been identified. However, only some are routinely used to choose the best treatment for affected patients [35]. In CMT, *BRCA1*, and *BRCA2* have, for example, already been investigated and were associated with an increased mammary tumor risk in English Springer Spaniels [36]. The analysis of such genes in CMT is nowadays still restricted to research purposes; therefore, the necessity to find novel markers to better assist the patients is noticeable [19]. The additional genes studied in this project were chosen based on the last findings on human breast cancer [18,36,37] and are believed to be worth investigating considering they may also play an important role in the tumor onset and development in dogs.

In this study, a considerably larger number of samples was analyzed when compared to other studies performed in this field so far, permitting, consequently, a more reliable outcome. The b-DNA assay permits the analysis of gene expression in canine mammary FFPE samples. Still, care should be taken considering that such specimens presented a generally lower expression of the analyzed target genes when compared to FF samples, a fact which might be related to the storage time. *ACTB* proved to be the best housekeeper to analyze gene expression in canine mammary tumors.

Gene expression studies in CMT are relevant regarding the fact that they can be applicable in practice just like in humans [18], helping the veterinarian to better assist the patient’s owner in making an appropriate approach as well as following the good breeding hygiene practice by correctly advising owners/breeders.

## 4. Materials and Methods

### 4.1. Tissue Samples

#### 4.1.1. Formalin-Fixed Paraffin Blocks

The specimens used in this study were retrospectively retrieved from the archives of the Pathology Institute of the University of Veterinary Medicine Hannover, Germany, between 1993 and 2000. The mammary samples were fixed in 10% neutral buffered formalin and routinely processed and embedded in paraffin wax. The blocks were stored at room temperature. A total number of 109 samples were analyzed, and 200 ng of RNA per sample were used for the measurement.

#### 4.1.2. Fresh Frozen Tumor Samples

The specimens were obtained from patients that had undergone surgery between 2003 and 2014 in the Clinic of Small Animals Hannover, Germany. The samples were frozen in liquid nitrogen immediately after surgical removal and maintained at −80 °C until RNA isolation. A total number of 93 samples were analyzed and 250 ng of RNA per sample were used for the measurement.

### 4.2. Nucleic Acid Isolation and Quantification

#### 4.2.1. Formalin-Fixed Paraffin Samples

For the FFPE blocks, prior to isolation of RNA, twice 20-μm-thick paraffin sections were prepared using a *microtome* (pfm Slide 2003, pfm medical ag, Cologne, Germany). Afterwards, the samples were deparaffinized, and subsequent RNA was isolated using the AllPrep DNA/RNA FFPE kit (Qiagen, Hilden, Germany) following the manufacturer’s instructions. The RNA yield was determined in the Synergy 2 reader (BioTek, Bad Friedrichshall, Germany). Samples were stored at −80 °C until use.

#### 4.2.2. Fresh Frozen Samples

Prior to the nucleic acid isolation, the frozen tissue was homogenized using a TissueLyser II—5 mm stainless steel bead (QIAGEN GmbH, Hilden, Germany). The nucleic acid isolation was performed with the RNAeasy mini kit (QIAGEN GmbH) following the manufacturer’s instruction. The RNA yield was determined in the Synergy 2 reader (BioTek, Bad Friedrichshall, Germany). Samples were stored at −80 °C until use.

#### 4.2.3. Target Genes

Sixteen (16) target genes (*Tp53*; *Myc*; *HMGA1*; *Pik3ca*; *Mcl1*; *MAPK3*; *FOXO3*; *PTEN*; *GATA4*; *PFDN5*; *HMGB1*; *MAPK1*; *BRCA2*; *BRCA1*; *HMGA2*; and *Her2*) acknowledged as having a certain influence on the onset and development of mammary tumors were tested for expression (details shown in Table 3).

#### 4.2.4. Housekeeping Genes

Three different housekeeping genes (HKG) commonly used as data normalizers for gene expression measurements in mammary tumors were used: *ACTB* (β-actin), *GAPDH* (Glyceraldehyde 3-phosphate dehydrogenase), and *HPRT1* (Hypoxanthine phosphoribosyltransferase 1).

#### 4.2.5. Luminex Technology (Branched-DNA (b-DNA) Assay)

The specimens were read using the Luminex^®^ 100/200™ System (Luminex Corporation, Austin, TX, USA) equipment which is bead-based, relying on fluorescent antibody-coupled beads detecting soluble analytes. The probe sets for the target genes were designed, based on their accession numbers (Table 3) by the assay provider AffymetrixeBioscience (Santa Clara, CA, USA).

Briefly, this technology works as follows: Each bead set is coated with a reagent specific to a particular target in order to detect a special analyte in the analyzed sample. Multiple readings are made on each bead set resulting in an individual fluorescent signal for each assay. The branched-DNA assay allows rapid and accurate analysis of up to 500 unique targets within a single sample (multiplexing).

During the early phase of the project, an evaluation of the technology on the biological material was performed in order to identify the ideal sample input for the specimens. The preliminary test showed that 250 ng of RNA for FF samples and 200 ng of RNA for FFPE specimens were sufficient amounts to perform the assay. Additionally, based on the amount of intra-assay consistency of duplicate measurements of certain samples observed during the experiments (represented by a low coefficient of variation), a single measurement was considered sufficient.

#### 4.2.6. Histological Classification

All the samples were checked and diagnosed by a pathologist, being based on hematoxylin and eosin staining and the nomenclature on Goldschmidt *et al.* [20]. For a more accurate and reliable analysis, the samples were allocated to small groups, which had at least 4 samples each. Some FFPE samples had eventually more than one diagnosis and were then not used in the analysis. The histological groups remained as indicated in Table 1.

#### 4.2.7. Data and Statistical Analysis

Prior to performing the statistical analysis, specimens with expression values below the LOD (limit of detection) were excluded (procedure recommended by the assay provider—AffymetrixeBioscience). The samples were normalized against the average of the three housekeeping genes and also independently normalized against each HKG. The results of double measurements intra-assay were averaged. Data were not normally distributed; therefore the Mann–Whitney *U* test was performed using the software SAS enterprise guide 5.1 (SAS Institute Inc., Cary, NC, USA). Values were considered statistically significant when *p* < 0.05.

#### 4.2.8. Effect of Storage Time

In order to assess the differences between the storage times of FFPE, samples were divided into three groups: 1. Storage time of 16 and 17 years, *n* = 74 (samples dated from 1999 and 2000); 2. Storage time of 18, 19, and 20 years, *n* = 61 (samples dated from 1996, 1997, and 1998); 3. Storage time of 21, 22, and 23 years, *n* = 30 (samples dated from 1993, 1994, and 1995). The data used for this analysis were those obtained directly after the branched-DNA assay (excluding the data below the LOD) before the normalization. Each target gene was tested in the three different storage time groups.

#### 4.2.9. Correlation between FF and FFPE

To assess the correlation between FF and FFPE, the median of the 10 histological groups for each target gene was calculated. Samples were taken from different animals; therefore, the correlation was performed as follows: TargetgeneFF *vs.* TargetgeneFFPE.

## Figures and Tables

**Table 1:** Histological groups based on Goldschmidt *et al.* [20] with their respective number of samples for FF and FFPE specimens. *n* ≥ 4.

Group	FF (*n*)	FFPE (*n*)
1. Healthy canine mammarytissue	13	4
2. Simple adenoma	6	4
3. Intraductal papillary adenoma	5	8
4. Complexa denoma	17	11
5. Benign mixed tumor	4	13
6. Simple tubular carcinoma	12	13
7. Solid carcinoma	8	7
8. Complex carcinoma	13	11
9. Ductal carcinoma	5	15
10. Carcinoma arising in a complex adenoma/mixed tumor	10	8

**Table 2:** Target genes and the histological groups: 1. healthy canine mammary tissue; 2. simple adenoma; 3. intraductal papillary adenoma; 4. complex adenoma; 5. benign mixed tumor; 6. simple tubular carcinoma; 7. solid carcinoma; 8. complex carcinoma; 9. ductal carcinoma and 10. carcinoma arising in a complex adenoma/mixed tumor. Asterisk (*) shows significant statistical differences (*p* < 0.05) between the two origins of samples (fresh frozen (FF) and formalin-fixed, paraffin-embedded (FFPE)) when analyzing the target genes within the histological groups. x represents *n* < 4 (data were not considered). – represents no statistically significant difference. Fifty-seven of 153 (37.25%) of the genes within their histological groups showed statistically significant difference.

Target Gene	1	2	3	4	5	6	7	8	9	10
*Tp53*	–	–	*	*	–	*	*	*	*	*
*Myc*	*	–	*	*	–	*	*	–	–	–
*HMGA1*	*	–	–	–	–	*	–	–	–	–
*Pik3ca*	–	–	–	–	–	–	–	–	–	–
*Mcl1*	*	–	–	–	–	*	–	–	–	*
*MAPK3*	–	–	–	*	–	*	–	–	*	*
*FOXO3*	–	–	–	–	–	–	–	–	–	*
*PTEN*	–	–	*	–	*	*	–	*	*	–
*GATA4*	–	x	–	–	*	*	–	–	–	x
*PFDN5*	–	–	–	–	–	–	–	–	–	*
*HMGB1*	*	*	*	*	*	*	*	*	*	*
*MAPK1*	*	*	*	*	–	*	*	*	*	*
*BRCA2*	–	x	–	*	–	–	*	–	–	–
*BRCA1*	–	x	x	–	–	–	–	–	–	–
*HMGA2*	*	–	x	*	*	*	x	–	–	–
*Her2*	–	–	–	–	–	*	*	–	–	–

**Table 3:** Genes with their respective accession numbers.

Gene	Accession Number
*BRCA1*	NM_001013416
*BRCA2*	NM_001006653
*FOXO3*	NM_003639400
*PDFN5*	NM_001251949
*PTEN*	NM_001003192
*Tp53*	NM_001003210
*GATA4*	NM_001048112
*Her2*	NM_001003217
*HMGA1*	NM_001003387
*HMGA2*	XM_005625590
*HMGB1*	NM_001002937
*MAPK1*	NM_001110800
*MAPK3*	NM_001252035
*Mcl1*	NM_001003016
*Myc*	NM_001003246
*Pik3ca*	XM_545208.4
*ACTB*	XM_536888
*GAPDH*	NM_001003142
*HPRT1*	NM_001003357
